# Fatigue Assessment of Twin Wire Arc Sprayed and Machine Hammer-Peened ZnAl4 Coatings on S355 JRC+C Substrate

**DOI:** 10.3390/ma15031182

**Published:** 2022-02-03

**Authors:** Michael P. Milz, Andreas Wirtz, Mohamed Abdulgader, Dirk Biermann, Wolfgang Tillmann, Frank Walther

**Affiliations:** 1 Materials Test Engineering (WPT), TU Dortmund University, Baroper Str. 303, 44227 Dortmund, Germany; frank.walther@tu-dortmund.de; 2 Institute of Machining Technology (ISF), TU Dortmund University, Baroper Str. 303, 44227 Dortmund, Germany; andreas.wirtz@tu-dortmund.de (A.W.); dirk.biermann@tu-dortmund.de (D.B.); 3 Materials Engineering (LWT), TU Dortmund University, Leonhard-Euler-Str. 2, 44227 Dortmund, Germany; mohamed.abdulgader@tu-dortmund.de (M.A.); wolfgang.tillmann@udo.edu (W.T.)

**Keywords:** twin wire arc spraying, machine hammer peening, coating, fatigue behavior, constant amplitude tests, corrosion protection, roughness

## Abstract

Structural elements for applications in maritime environments, especially offshore installations, are subjected to various stresses, such as mechanical loads caused by wind or waves and corrosive attacks, e.g., by seawater, mist and weather. Thermally sprayed ZnAl coatings are often used for maritime applications, mainly due to good corrosion protection properties. Machine hammer peening (MHP) has the potential to increase fatigue and corrosion fatigue resistance of ZnAl coatings by adjusting various material properties such as hardness, porosity and roughness. This study investigates the fatigue behavior of twin wire arc sprayed and MHP post-treated ZnAl4 coatings. Unalloyed steel (S355 JRC+C) was selected as substrate material and tested as a reference. MHP achieved the desired improvements in material properties with increased hardness, decreased roughness and uniform coating thickness. Multiple and constant amplitude tests have been carried out to evaluate the fatigue capability of coating systems. In the high cycle fatigue regime, the additional MHP post-treatment led to an improvement of the lifetime in comparison to pure sandblasted specimens. The surface was identified as a crack initiation point. ZnAl coating and MHP post-treatment are suitable to improve the fatigue behavior in the high cycle fatigue regime compared to uncoated specimens.

## 1. Introduction

Structural elements are often made of unalloyed steels, particularly due to relatively low costs and good weldability. For applications in marine environments, e.g., offshore wind turbine towers or bridges, coatings are necessary for corrosion protection. Metallic coatings work as a passive barrier and active cathodic corrosion protection. The key element in the selection of suitable coating systems for active cathodic protection is the electrode potential. As the used metallic coatings have a more negative electrode potential than steel, the substrate material and the coating form a galvanic couple. Consequently, the coating acts as a sacrificial anode and degrades preferably while the substrate is protected. In the case of ZnAl coatings, the corrosion products also increase the corrosion resistance as they act as a barrier between the coating and electrolyte [1].

In service, structural elements in marine environments are subject to corrosive stresses as well as various mechanical stresses, e.g., due to wind, waves or tide. In order to withstand these superimposed mechanical, corrosive and environmental stresses over many years, a combination of anti-corrosion coatings, organic coatings such as paintings, sacrificial anode protection and impressed current cathodic protection are used [2]. For structural elements in a marine environment, ZnAl-based coatings are well established [3]. It has been shown that Aluminum and alloying elements such as Manganese or Silicon can be added to improve the corrosion resistance of pure Zinc coatings [4,5,6].

Galvanization or thermal spraying processes are used to apply ZnAl-based coatings. Disadvantages of galvanization compared to thermal spraying processes are the limitation of the component dimensions to the size of the used electroplating as well as the formation of brittle Fe-Zn intermetallic phases [1], which are known to reduce the mechanical performance. In comparison, thermal spraying introduces less heat during the process and is less limited in terms of component dimensions [7]. Nevertheless, thermal sprayed coatings have higher porosity than galvanic coatings, a lamellar structure and thermally or kinetically induced residual stresses [8]. Compressive residual stresses are beneficial and increase the fatigue strength of components, while tensile residual stresses lead to degradation [9]. Higher temperatures in the coating process result in tensile residual stresses, while processes with higher kinetic energy lead to compressive residual stresses [10]. It has been shown that compressive residual stresses were present in ZnAl coatings produced by the twin wire arc spraying (TWAS) process as an effect of the kinetic energy caused by larger and not completely melted impacting particles [11].

In addition to the organic coatings, anode protection and current cathodic protection [2], mechanical post-treatment of the coating, e.g., machine hammer peening (MHP) or shot peening and thermo-mechanical post-treatments such as hot isostatic pressing (HIP), can be carried out [12]. The positive effect of mechanical post-treatment on corrosion behavior is based on filling pores, eliminating porosity, densifying, and homogenizing the coating structure [12]. Mechanical reworking by means of the MHP process leads to a decrease in porosity, an increase in hardness and the induction of compressive residual stresses in the near-surface area [13,14]. This relationship was also found for ZnAl-coatings [11,15]. Wirtz et al. have shown that a temperature superimposed MHP process can lead to an increase of densification, hardness and induced compressive residual stresses for ZnAl-based coatings compared to MHP processes performed at room temperature [16]. The high potential of MHP post-treated ZnAl coatings for improvement of the fatigue and corrosion fatigue behavior was not investigated until now.

In the field of tool and mold making, it has already been exhibited for conventional uncoated tool systems that piezo peening, which can be assigned to MHP processes, can be used to smooth the surface. Furthermore, compressive residual stresses and work hardening induced by MHP can lead to an increase in tool life [17]. Branci et al. investigated the fatigue behavior of welded joints that had been repaired with MHP after pre-fatigue loading. Cracks had already formed due to pre-loading [18]. The fatigue life of the repaired welded joints was significantly increased compared to the unrepaired joints and the undamaged joints [18]. Punchi-Cabrera et al. [19] investigated the fatigue behavior of SAE 1045 steel coated with a NiCrBSiW (Colmonoy 88) coating. Fatigue tests in air showed a decrease in fatigue life of coated specimens compared to polished uncoated specimens. In contrast, the additional coating led to an improvement in corrosion fatigue life in NaCl solution compared to uncoated specimens.

In this study, the fatigue behavior of the ZnAl4-coating systems deposited on the unalloyed steel S355 JRC+C was investigated to evaluate the influence of the coating and mechanical post-treatment (MHP) with the aim to transfer the results to industrial marine applications, e.g., wind turbine towers. For the fatigue assessment coupled multiple amplitude tests (MAT) and constant amplitude tests (CAT) were carried out. Mechanical, thermal and electrical measurement technologies were applied simultaneously to evaluate and compare the material deformation and damage evolution, e.g., plastic deformation and crack initiation during cyclic loading.

## 2. Materials and Methods

### 2.1. Materials and Manufacturing

**Figure 1:**
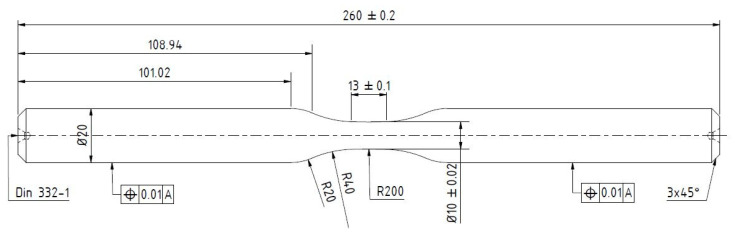
Specimen geometry.

Three different material conditions, I-III, based on ZnAl4-coating on unalloyed structural steel S355 JRC+C (1.0551) substrate were investigated. Therefore, specimens according to the geometry in Figure 1, the substrates were sandblasted (I), I + ZnAl4-coated (II) and II + MHP post-treated (III).

**Figure 2:**
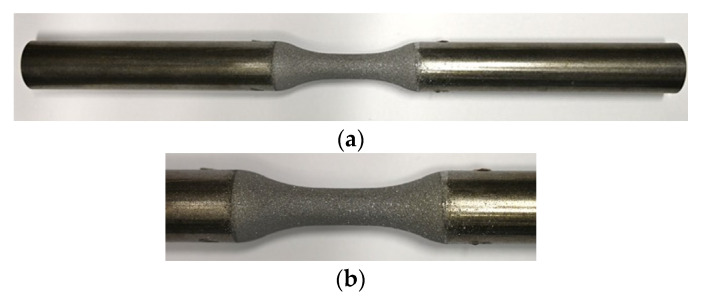
Macro images of sandblasted (I) specimen, (**a**) overview, (**b**) detail of gauge length.

After S355 JRC+C machining, the surfaces of the gauge length were sandblasted to ensure sufficient coating adhesion. Sandblasting was carried out using Alumina oxide powder with EKF 24 (−850 µm + 600 µm) size fraction, a blasting pressure of 4 bar, 100 mm stand-off distance and a blasting angle of 45°. After sandblasting, an ultrasonic Ethanol bath was used to remove the dust residues and clean up the sandblasted surfaces. An S355 JRC+C specimen in sandblasted condition (I) is shown in Figure 2. Surface treatments are known to affect fatigue behavior due to induced hardening, residual stresses and higher roughness. Therefore, sandblasted (I) samples were chosen as a reference to ensure comparability with coating systems (II-III), which were sandblasted before coating in this study.

#### 2.1.1. Thermal Spray Process

The coating process was performed within the gauge length and diameter of 10 mm exclusively. The surface of specimens was cleaned using Ethanol prior to coating to remove contaminations such as residues of oil. The twin-wire arc spraying (TWAS) process was used to apply ZnAl4 coatings on the sandblasted specimens’ surface. The spray unit was Durum Duraspray 450 (Durum, Germany). The chemical composition of the feedstock wires made of ZnAl4 is shown in Table 1.

**Figure 3:**
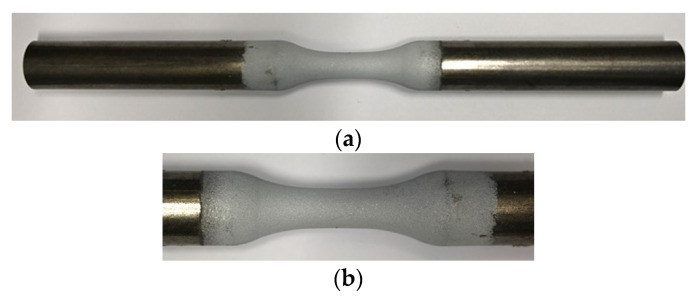
Macro images of ZnAl-coated (II) specimen, (**a**) overview, (**b**) detail of gauge length.

In the experiments, dry and compressed air was utilized as atomization gas. The adjusted process parameters were 3.2 m/min wire feedrate, 22 V arc voltage and 5 bar atomization gas pressure. The coating process was carried out using an industrial robot ABB IRB 4600 and a rotating unit. The handling parameters were stand-off distance between the spray gun and substrate surface of 120 mm, axial gun velocity vs = 18,000 mm/min, and meander spacing s = 4 mm. All coatings were made in four gun overruns in total. Masking of uninvolved areas was applied during the spraying process to avoid unwanted coating of these areas. The coating thickness was chosen based on offshore applications and should be at least as thick as required by standards. In DIN EN ISO 206 [20] a coating thickness of 200 µm is proposed for spray coatings with a service life of 20 years for very severe corrosivity in coastal atmospheres with high saltwater exposure (corrosivity category C5 according to DIN EN ISO 12944 [21]). Figure 3 shows a ZnAl-coated (II) specimen.

#### 2.1.2. Mechanical Post-Treatment

**Figure 4:**
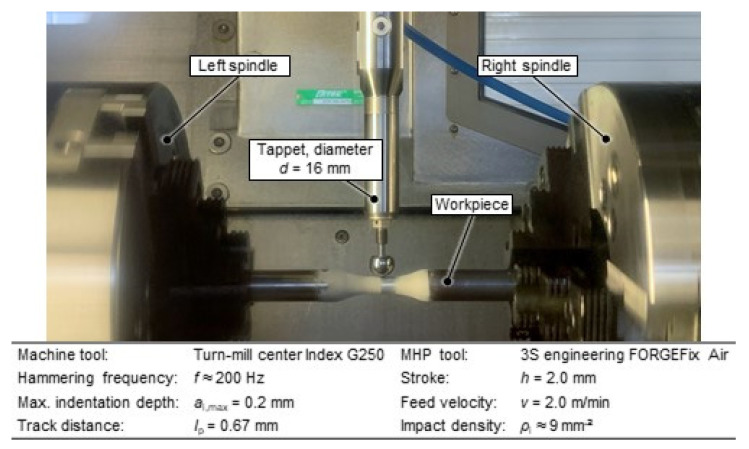
Turn-mill center with process parameters for machine hammer peening (MHP) process.

After ZnAl-coating, the MHP process was applied with the aim of improving the fatigue properties. By mechanical compacting of the coating, a reduction of porosity and roughness, as well as an increase of compressive residual stresses and hardness, is expected. The coated specimens were peened using a high-performance turn-mill center Index G250 with a 3 s engineering FORGEFix Air MHP tool using a solid carbide ball with diameter *d*_p_ = 16 mm (Figure 4).

**Figure 5:**
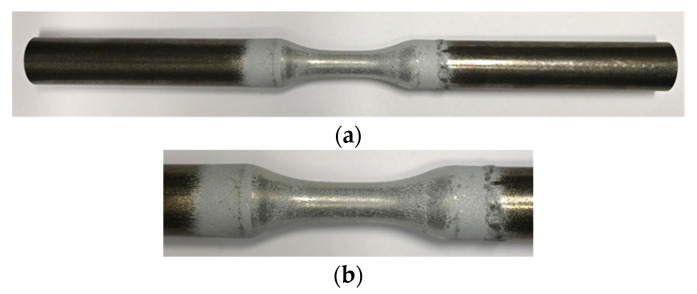
Macro images of ZnAl-coated and machine hammer-peened (III) specimen, (**a**) overview, (**b**) detail of gauge length.

Based on former studies [15,22], the process parameter settings were selected and kept constant. A compressed air pressure *p* = 6 bar resulted in a hammering frequency *f* ≈ 200 Hz with a stroke set to *h* = 2.0 mm. A feed speed *v*_f_ = 2.0 m/min, a maximal indentation depth *a_i,max_* ≈ 0.2 mm and a line pitch *l*_p_ = 0.67 mm were used. The process parameter values resulted in an impact density of about *p*_i_ = 9 indents/mm^2^. The NC path along the workpiece surface was generated with a maximum indentation depth *a_i,max_* = 0.2 mm for all process variants by using computer-aided machining software. Figure 5 shows an MHP post-treated (III) specimen.

### 2.2. Material Characterization and Testing Methods

#### 2.2.1. Macro- and Microstructure Investigations

Material characterizations of the three conditions, I-III, were applied to evaluate the influence of coating and post-treatment on surface quality. Coating thickness, mean roughness depth R_z_, arithmetical mean roughness R_a_, mean smoothing depth R_p_ and coating hardness characterized before and after MHP post-treatment. The roughness parameters were determined using a confocal white light microscope Nanofocus μsurf with magnification 50× long objective and robust Gaussian filter with cutoff wavelength λ_c_ = 0.25 mm (R_z,_ and R_a_) and λ_c_ = 0.8 mm (R_p_). Furthermore, cross-sections were prepared metallographically in order to determine the coating and substrate morphology using Olympus BX51 optical microscope and Olympus stream motion software.

#### 2.2.2. Mechanical Investigations

Tensile and fatigue tests were carried out according to ISO 6892-1 using a servohydraulic testing system (Instron, 8802, F = ±250 kN). In contrast to standardized tensile specimens, the geometry has a reduced gauge length of 12 mm. The tests were performed at a constant strain rate $\dot{\varepsilon_{c}}$ = 0.0007 s^−1^ up to a total strain of 8%, afterwards, the extensometer was removed, and the tests were controlled by crosshead speed v_c_ = 0.00325 mm/s until failure. A minimum of two tensile tests for each condition I-III were performed.

Multiple amplitude tests (MAT) and constant amplitude tests (CAT) were carried out for fatigue characterization. Fatigue load was applied by using a sinusoidal tension-compression load-time function at a stress ratio R = −1 (fully-reversed loading). MAT were conducted with a starting maximum stress σ_a,start_ = 100 MPa, followed by a stepwise increase of Δσ_a_ = 10 MPa per ΔN = 10^4^ cycles, until failure. Based on MAT results the stress amplitudes 280, 320 and 360 MPa were determined for CAT until a limited number of cycles N_limit_ = 2 × 10^6^. Stresses were calculated based on the initial diameter of sandblasted S355 JRC+C specimens, even for coated specimens. The assumption of the diameter including the coating would lead to higher stresses than intended.

**Figure 6:**
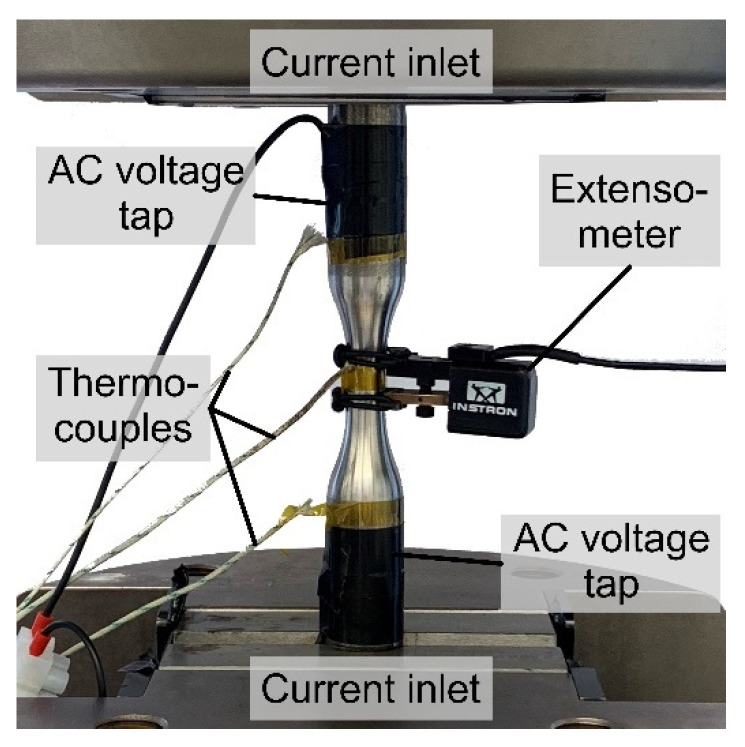
Experimental setup for mechanical testing.

The test program including MAT and CAT was carried out with the same testing and measurement setup consisting of an extensometer with 10 mm gauge length, four thermocouples and alternating current potential drop (ACPD) technology (Figure 6). The thermocouples were located in the middle of the gauge length and at the shafts. In addition, a thermocouple was attached to a specimen near the testing system to record ambient temperature changes for compensation issues. The alternating current was injected via copper plates below the clamping jaws and tapped via welded cables on the shafts, whereas the specimen and clamping were electrically isolated from the testing system. When using ACPD application at a frequency of 1000 Hz, due to skin effect, near-surface region of a few hundred micrometers is analyzed, allowing high-precise consideration of surface changes.

## 3. Results and Discussion

### 3.1. Substrate Material and Coating Systems

**Figure 7:**
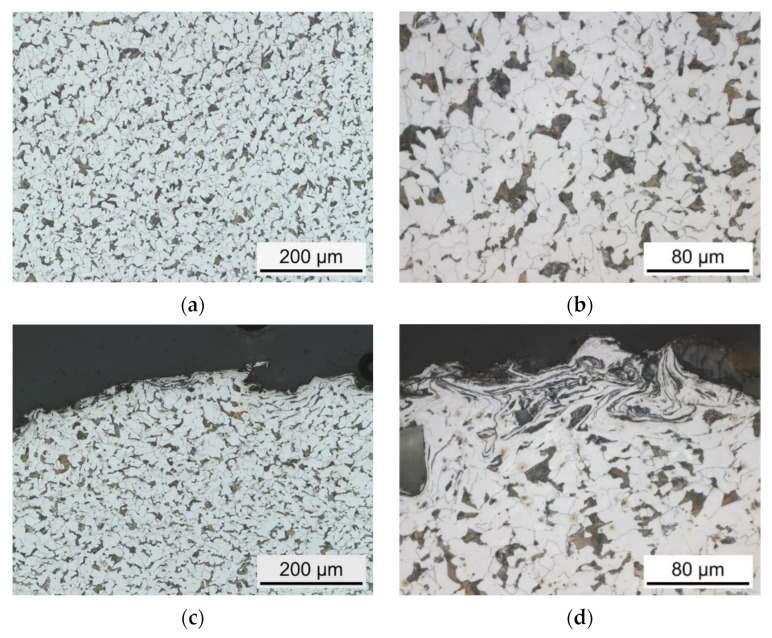
Polished cross-section of sandblasted (I) specimen; etched with 5% Nital solution, (**a**,**b**) microstructure of center area, (**c**,**d**) microstructure of edge area.

The substrate S355 JRC+C steel has a ferritic-pearlitic microstructure, shown in Figure 7. All specimens were etched with 5% Nital. White areas are ferritic and dark areas are pearlitic.

**Figure 8:**
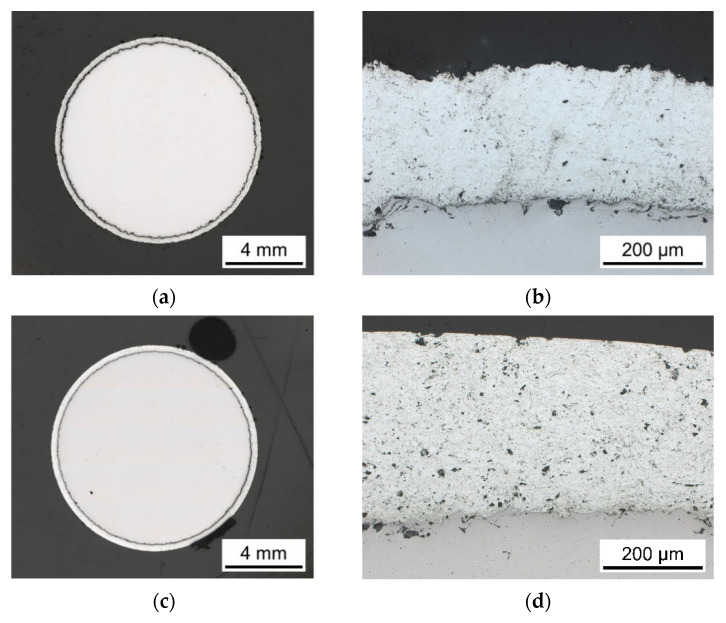
Images of coating systems, (**a**) overview of ZnAl-coated (II) specimen, (**b**) ZnAl-coating (II), (**c**) overview of machine hammer-peened (III) specimen, (**d**) machine hammer-peened (III) ZnAl-coating.

Figure 8 shows the cross-sections through the gauge length of ZnAl-coated (II), and MHP post-treated (III) specimens. Both coating systems do not reveal any gaps or cracks, Figure 8a,c. Figure 8b,d displays a continuous layer, while MHP post-treated (III) coating has a smoother surface than ZnAl-coating (II), which was confirmed by roughness measurements.

Light microscopic images were taken at three locations to determine the coating thickness. Table 2 shows the coating thickness as average, including the standard deviation of three measurements at three locations. After MHP post-treatment (III), standard deviations of coating thickness are significantly lower than after coating (II). The higher average value of III compared to II can only be due to thickness deviations in the spraying process. Thus, the requirement for a coating thickness higher than 200 µm according to DIN EN ISO 206 [20] was successfully achieved. In addition, the images show a smoother surface, lower porosity and improved interfacial bonding for MHP post-treated (III) coating compared to untreated (I) coating.

Three macro hardness measurements were carried out in the center of the substrate, leading to 194 ± 2 HV10. The microhardness determined by three measurements results in 22 ± 2 HV0.05 for ZnAl-coating (II) and 28 ± 3 HV0.05 for MHP post-treated (III) coating, i.e., an increase of 22%.

**Figure 9:**
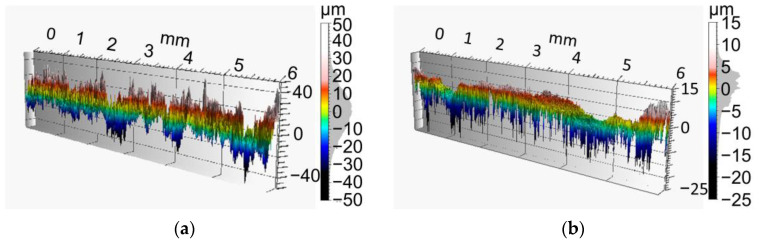
Surface profiles of coating systems: (**a**) ZnAl-coated (II) specimen, (**b**) machine hammer-peened (III) specimen.

Figure 9 shows roughness values as profiles for ZnAl-coated (II) and MHP post-treated (III) specimens, whereas two specimens per condition were analyzed for 6 mm length, shown in Table 3.

Highest values were evaluated for the sandblasted (I) condition to improve coating adhesion. MHP post-treatment after coating process (III) results in the lowest roughness values and ZnAl-coating (II) ranges in between. Comparing the average roughness $\bar{R}$_p,coated_ = 29.1 µm and $\bar{R}$_p,MHP_ = 15.6 µm, a reduction of coating up to 46% by MHP can be stated.

### 3.2. Quasistatic Deformation Behavior

**Figure 10:**
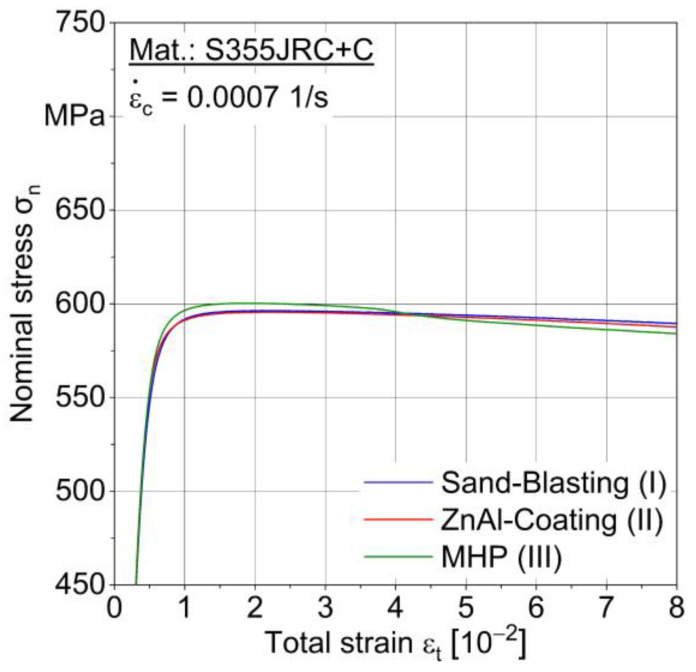
Stress-strain curves for sandblasted (I), ZnAl-coated (II) and machine hammer-peened (III) specimens.

Figure 10 shows stress-strain (σ-ε) curves for conditions I–III until 8% total strain. Tensile strength from 596 to 622 MPa and yield stress from 560 to 590 MPa were obtained, see Table 4. A significant influence of the ZnAl-coating and MHP process on the quasi-static material properties could not be determined.

### 3.3. Cyclic Deformation Behavior

#### 3.3.1. Multiple Amplitude Tests (MAT)

**Figure 11:**
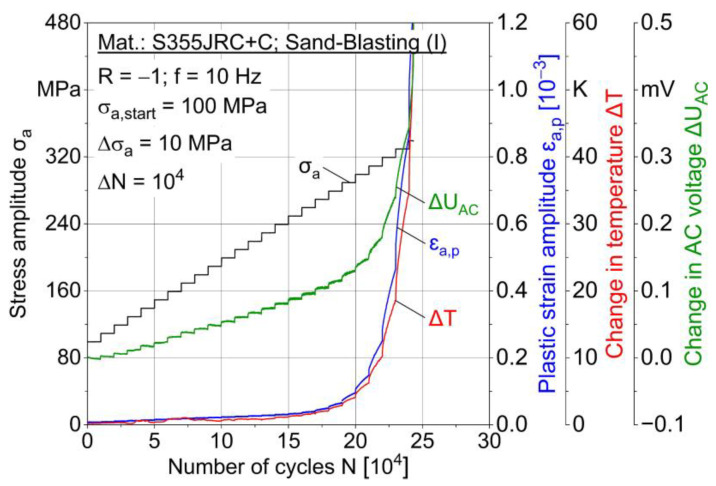
Multiple amplitude test (MAT) for sandblasted (I) specimen.

**Figure 12:**
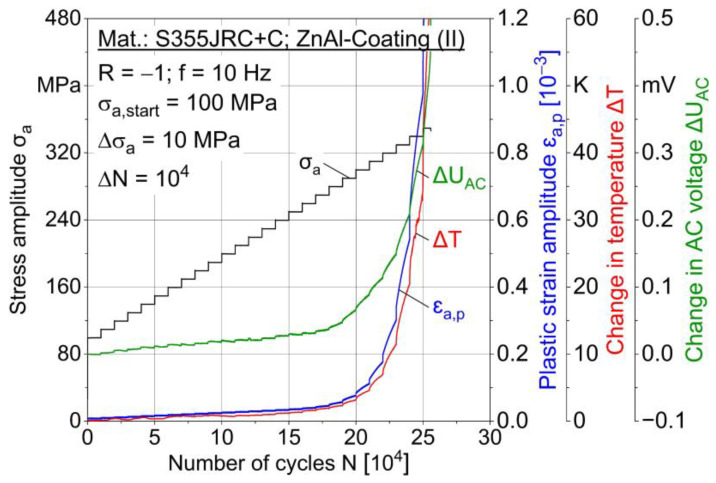
Multiple amplitude test (MAT) for ZnAl-coated (II) specimen.

**Figure 13:**
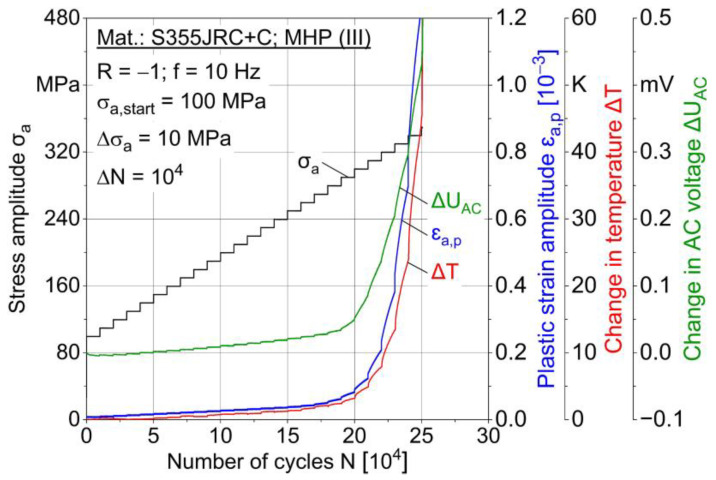
Multiple amplitude test (MAT) for MHP post-treated (III) specimen.

In multiple amplitude tests [23], the following material reaction parameters are evaluated: Plastic strain amplitude ε_a,p_, loading-induced change in temperature ∆T and change in AC voltage ∆U_AC_ plotted as the number of cycles in dependence independent of the stepwise load increase. Two MAT were carried out for each condition, whereas one characteristic test result is shown in Figure 11, Figure 12 and Figure 13 for conditions I–III.

A correlation of the measured values is obvious, e.g., between plastic strain and temperature. Increasing AC voltage change is expected to result from loading-induced material changes, such as microcracks and temperature increase due to plastic strain. Both sandblasted (I) specimens failed at the stress amplitude 340 MPa, while MHP post-treated (III) specimens failed at 350 MPa and ZnAl-coated (II) specimens at 340 and 350 MPa. MAT results indicate a better fatigue capability of ZnAl-coated (II) and post-treated (III) specimens compared to sandblasting (I). For ∆T and ∆U_AC_ results of MAT and CAT, a median filter with factor 20 was applied.

**Figure 14:**
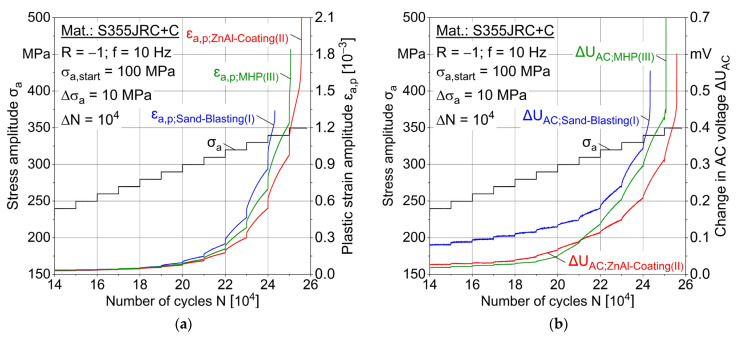
Plastic strain amplitude and change in AC voltage for multiple amplitude test (MAT) for sand-blasting (I), ZnAl-coating (II) and machine hammer peening (MHP) (III), comparison of (**a**) plastic strain amplitude, (**b**) change in AC voltage.

Figure 14 summarizes the conditions I-III, the ε_a,p_ and ∆U_AC_ results as a function of loading cycles. The first significant changes in plastic strain amplitude within the same load level are seen after 18 × 10^4^ cycles shown in Figure 14a. The first changes in AC voltage in the same lifetime region are shown in Figure 14b.

Based on these MAT results, stress amplitudes for constant amplitude tests (CAT) were determined. The first stress amplitude was selected to be σ_a_ = 360 MPa. At this level, failure is expected in the low cycle fatigue (LCF) regime. The second stress amplitude was chosen by considering the first material changes, which are σ_a_ = 280 MPa. At this level, failure is expected in the high cycle fatigue (HCF) regime. The third stress amplitude was set in between, i.e., σ_a_ = 320 MPa.

#### 3.3.2. Constant Amplitude Tests (CAT)

**Figure 15:**
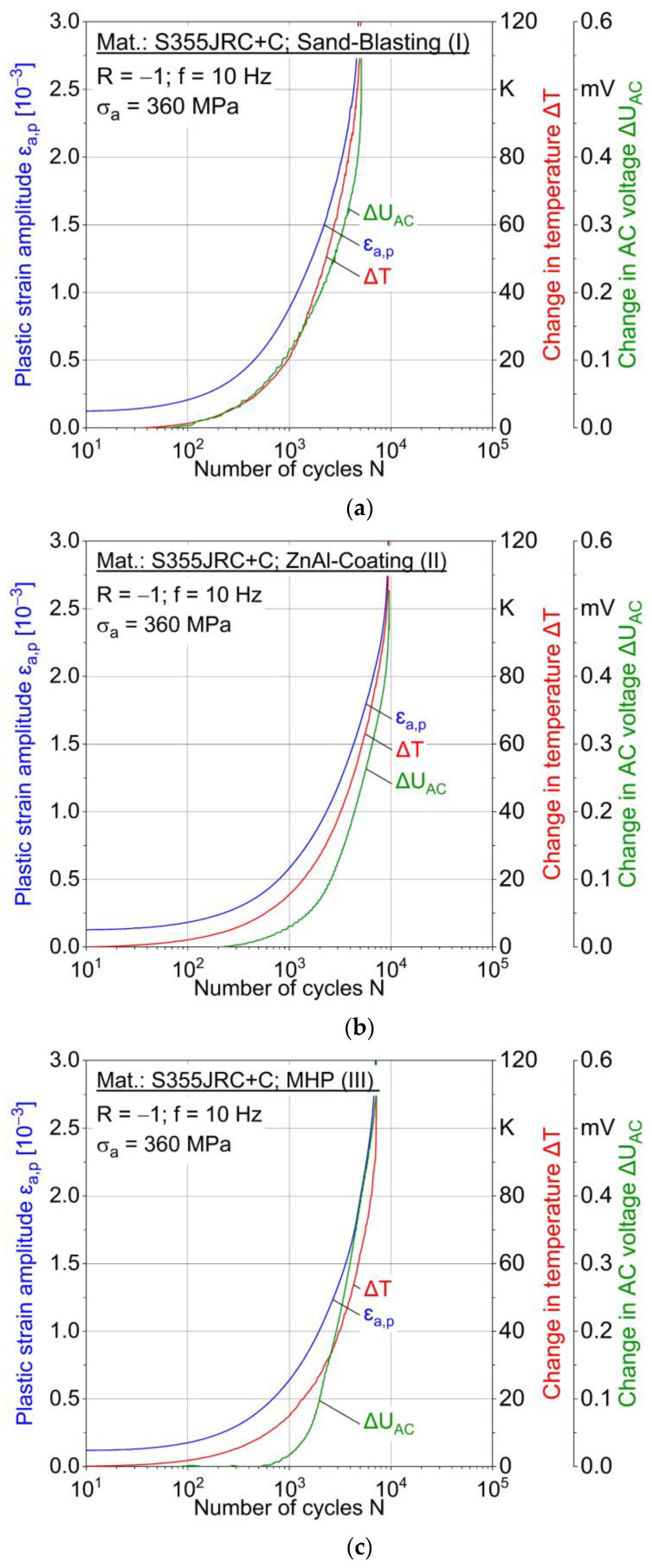
Constant amplitude tests (CAT) at 360 MPa for (**a**) sandblasted (I), (**b**) ZnAl-coated (II) and (**c**) machine hammer-peened (III) specimens.

**Figure 16:**
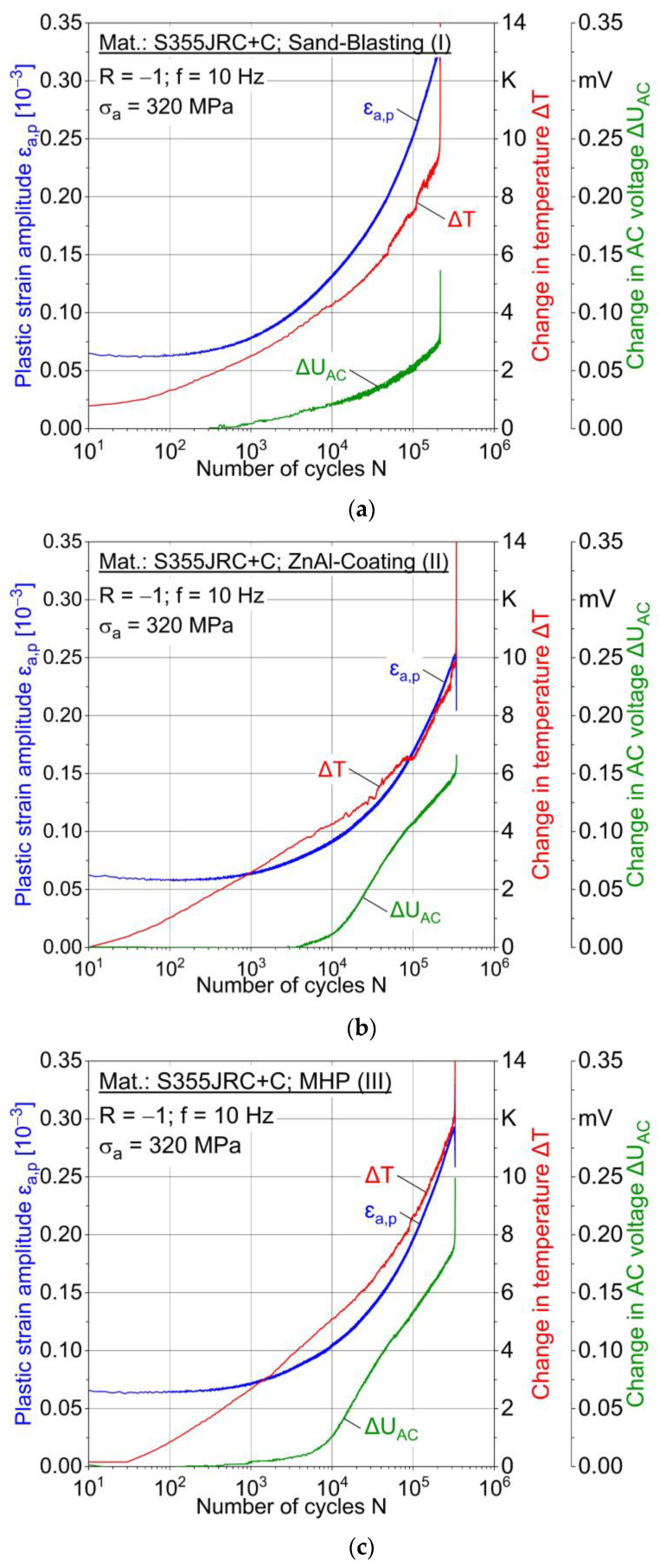
Constant amplitude tests (CAT) at 320 MPa for (**a**) sandblasted (I), (**b**) ZnAl-coated (II) and (**c**) machine hammer-peened (III) specimens.

**Figure 17:**
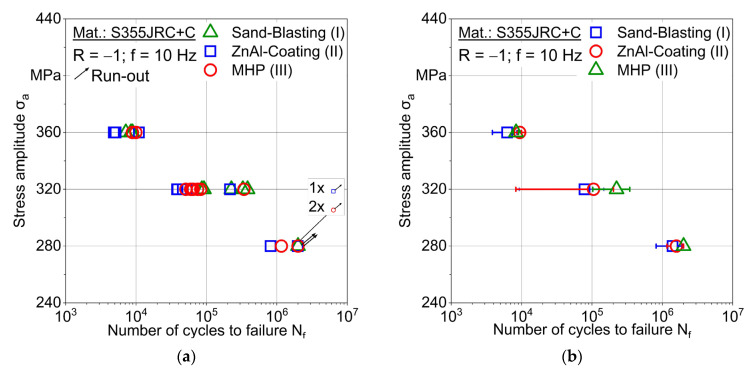
S-N diagrams of conditions sand-blasting (I), ZnAl-coating (II) and machine hammer-peened (III), (**a**) individual lifetime values, (**b**) average lifetime values with standard deviations.

**Figure 18:**
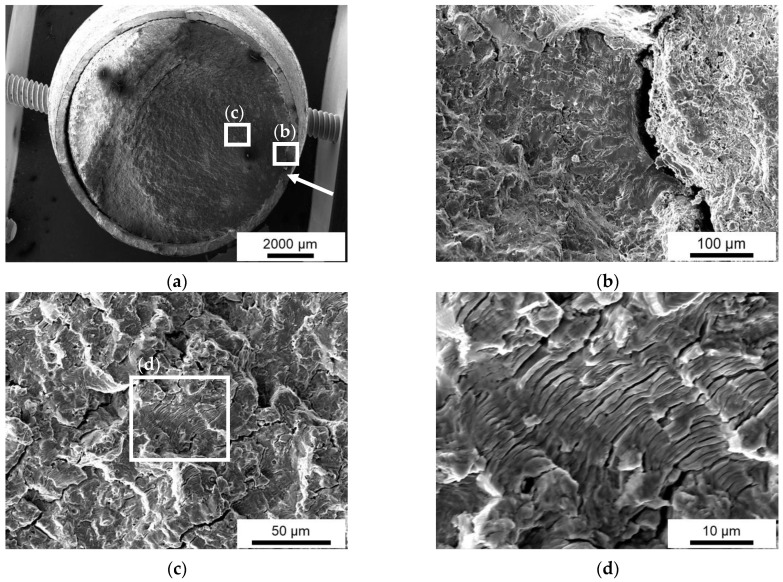
Fractography images of machine hammer-peened (III) specimen loaded at 320 MPa, (**a**) overview, (**b**) crack initiation from edge, (**c**) fatigue fracture area, (**d**) fatigue striations.

Two constant amplitude tests were carried out for each stress amplitude and condition I-III. Figure 15 shows the results for 360 MPa and Figure 16 for 320 MPa in the form of cyclic deformation curves, i.e., material reaction as f (cycles). For σ_a_ = 360 MPa, the behavior of the conditions I-III is characterized by pure cyclic softening from the beginning until failure. In Figure 15a, delayed change in AC voltage compared to change in temperature and plastic strain amplitude is observed. For MHP post-treated (III) specimens, a plastic strain amplitude of up to 0.4% (0.03%) was obtained at a stress amplitude of 360 MPa (320 MPa). The higher plastic deformation at 360 MPa results in higher temperature changes. MHP post-treated (III) specimen shows a temperature increase of 120 K at 360 MPa just before failure (Figure 17), while it is only 12 K at 320 MPa (Figure 18).

Figure 17a shows an S-N-diagram with stress amplitudes plotted versus lifetime, i.e., number of cycles to failure. At 360 MPa, specimens failed between 0.48 × 10^4^ and 1.10 × 10^4^ cycles. At 320 and 280 MPa, a higher scatter band leading to a greater lifetime range is determined. Nevertheless, MHP post-treatment (III) tends to improve fatigue performance. For 280 MPa, all MHP post-treated (III) specimens lead to run-outs, while sandblasted (I) specimens do not. At the same stress amplitude, only one ZnAl-coated (II) specimen achieved a run-out. Fatigue stresses were calculated based on the initial diameter of sandblasted S355 JRC+C specimens in order to ensure the comparability between coated (II), MHP post-treated (III) and sandblasted (I) specimens. Run-out specimens showed numerous cracks within the coating without specimen failure. Since the coating fails early during fatigue loading and provides no force bearing capacity, mechanical stresses need to be calculated only based on the initial cross-sections of sandblasted specimens. The comparable progression of quasi-static stress-strain curves of the conditions I-III also supports the procedure (Figure 10).

The S-N-presentation with stress amplitudes plotted versus the average value of the number of cycles to failure, including standard deviation, improves the possibilities of fatigue assessment regarding optimized process condition selection, as shown in Figure 17b. ZnAl-coating (II) and MHP post-treatment (III) improve the fatigue behavior leading to greater lifetimes at the same fatigue loading level. Consequently, differences in the porosity of the coating and interfacial bonding due to the MHP process (Figure 8) do not influence the tensile and fatigue behavior. The stress amplitudes selected for S-N curves based on a minimum specimen number in terms of an efficient testing strategy provide a good indication of the fatigue performance of the conditions sandblasted (I), ZnAl-coated (II) and machine hammer-peened (III).

Scanning electron microscopy (SEM) investigations showed similar fracture mechanisms for the conditions I-III. In Figure 18a, the MHP post-treated (III) specimen loaded at 320 MPa was analyzed. The crack started at the surface, and from there a fatigue fracture propagated through the volume until sudden failure. The visual examinations of the run-out conditions (II-III), where cracks have already been detected in the surface, confirm the observations. SEM images allow a clear separation of the fatigue fractures and residual force fractures. Fatigue fraction area increases with decreasing stress amplitude.

About two-thirds of the fracture area is indicated as a fatigue area. The white arrow marks the crack initiation as the starting point for fatigue crack propagation, shown in Figure 18a. Figure 18b shows a location of crack initiation from the surface through the ZnAl coating. The fatigue fracture surface is characterized by side cracks propagating along fatigue lines, and between fatigue striations are clearly visible, shown in Figure 18c,d.

## 4. Conclusions and Outlook

Using the twin-wire arc spraying (TWAS) process, a covering ZnAl4 coating with a thickness above 200 µm was produced on an S355 JRC+C substrate. Machine hammer peening (MHP) improved the material properties such as hardness and roughness, which resulted in better fatigue performance determined in multiple and constant amplitude tests. In addition, a coating thickness with less scatter could be received with the MHP process. ZnAl-coated (II) and MHP post-treated (III) specimens exhibited better fatigue behavior than sandblasted (I) specimens for the investigated loading range from 280 to 360 MPa stress amplitude. Thus, ZnAl4 coating and MHP post-treatment after ZnAl4 coating are suitable to improve the fatigue performance in the high cycle fatigue (HCF) regime. Constant amplitude tests determined that plastic strain amplitudes correlate with changes in temperature and changes in AC voltage, i.e., all microstructure-sensitive measurement technologies are capable of determining the current fatigue state and loading-dependent fatigue evolution. Crack initiation starts from the surface and cracks propagate through the material volume until sudden failure.

Further studies at higher frequencies in ACPD application will improve the evaluation of near-surface changes, whereas iterative experiments will be used to quantify the effects of the temperature on the measured voltage. Additionally, the aspect of corrosion will be investigated in the form of polarization tests and corrosion fatigue tests, as ZnAl coatings are often used in a corrosive environment.

## Figures and Tables

**Table 1:** Chemical composition of ZnAl4 feedstock wires according to the manufacturer datasheet.

Element	Zn	Al	Si	Fe	Pb	Cu	Sn
wt.-%	Bal.	3.5–4.5	≤0.030	≤0.005	≤0.003	≤0.002	≤0.001

**Table 2:** Average coating thickness for three measurements each, at three locations in gauge length center: ZnAl-coated (II) and machine hammer-peened (III) specimens.

Specimen Surface	Coating Thickness in µm
ZnAl-coated (II)	244.0 ± 23.5|284.6 ± 39.9|281.9 ± 50.3
Machine hammer-peened (III)	319.5 ± 21.1|330.4 ± 7.2|337.9 ± 5.8

**Table 3:** Roughness values in gauge length as axial line scan, for two specimens each: Sandblasted (I), ZnAl-coated (II), and machine hammer-peened (III) specimens.

Specimen Surface	Mean Smoothing Depth Rp in µm	Mean Roughness Depth Rz in µm	Arithm. Mean Roughness Ra in µm
Sandblasted (I)	29.6 ± 3.3|40.4 ± 6.3	29.3 ± 2.7|35.7 ± 1.7	5.5 ± 0.5|6.0 ± 0.4
ZnAl-coated (II)	22.2 ± 2.1|25.9 ± 2.4	26.9 ± 1.4|31.2 ± 2.1	5.1 ± 0.3|5.4 ± 0.3
Machine hammer-peened (III)	4.2 ± 0.7|14.0 ± 3.5	11.6 ± 1.0|19.7 ± 2.3	1.6 ± 0.2|3.1 ± 0.4

**Table 4:** Average tensile properties for two specimens each: Sandblasted (I), ZnAl-coated (II) and machine hammer-peened (III) specimens.

Specimen Condition	Tensile Strength Rm in MPa	Yield Strength Rp 0.2 in MPa
Sandblasted (I)	597 ± 1	561 ± 1
ZnAl-coated (II)	601 ± 5	571 ± 4
Machine hammer-peened (III)	611 ± 11	580 ± 10

## Data Availability

Not applicable.
